# Turtle Insights into the Evolution of the Reptilian Karyotype and the Genomic Architecture of Sex Determination

**DOI:** 10.3390/genes11040416

**Published:** 2020-04-11

**Authors:** Basanta Bista, Nicole Valenzuela

**Affiliations:** Department of Ecology, Evolution, and Organismal Biology, Iowa State University, Ames, IA 50011, USA

**Keywords:** sex chromosome evolution, karyotypic and molecular evolution, genomic architecture of sexual development, adaptation and natural selection, genome organization and function, nucleolar organizing region, dosage compensation, faster-X and faster-Z, climate change and global warming, reptilian vertebrates

## Abstract

Sex chromosome evolution remains an evolutionary puzzle despite its importance in understanding sexual development and genome evolution. The seemingly random distribution of sex-determining systems in reptiles offers a unique opportunity to study sex chromosome evolution not afforded by mammals or birds. These reptilian systems derive from multiple transitions in sex determination, some independent, some convergent, that lead to the birth and death of sex chromosomes in various lineages. Here we focus on turtles, an emerging model group with growing genomic resources. We review karyotypic changes that accompanied the evolution of chromosomal systems of genotypic sex determination (GSD) in chelonians from systems under the control of environmental temperature (TSD). These transitions gave rise to 31 GSD species identified thus far (out of 101 turtles with known sex determination), 27 with a characterized sex chromosome system (13 of those karyotypically). These sex chromosomes are varied in terms of the ancestral autosome they co-opted and thus in their homology, as well as in their size (some are macro-, some are micro-chromosomes), heterogamety (some are XX/XY, some ZZ/ZW), dimorphism (some are virtually homomorphic, some heteromorphic with larger-X, larger W, or smaller-Y), age (the oldest system could be ~195 My old and the youngest < 25 My old). Combined, all data indicate that turtles follow some tenets of classic theoretical models of sex chromosome evolution while countering others. Finally, although the study of dosage compensation and molecular divergence of turtle sex chromosomes has lagged behind research on other aspects of their evolution, this gap is rapidly decreasing with the acceleration of ongoing research and growing genomic resources in this group.

## 1. Introduction

A paramount event in the history of life is the early evolution of sexual reproduction. Sex, which is nearly universal in eukaryotes, joins half of two parental genomes from gametes produced by meiosis into unique combinations, contributing to the phenotypic diversity that is naturally selected during adaptive evolution [1]. However, the evolution of gametes into two types (small and mobile versus large and immobile) that led to the evolution of male and female functions meant that a developmental decision is necessary for individuals to become sperm-producing males, egg-producing females, or hermaphrodites that produce both. In multicellular organisms, where separated sexes evolved often, and particularly in animals, this decision is controlled most frequently by a pair of specialized chromosomes (the sex chromosomes) [2]. In other animals, this decision is plastic and occurs in response to environmental cues (ESD), of which the most common in vertebrates is temperature (TSD) [3]. However, a clear explanation for why some vertebrate lineages rely exclusively on sex chromosomes, some exclusively on TSD, and some combine both [4,5,6], remains elusive, as are the mechanistic trajectories by which species transition between TSD and sex chromosomes. Reptiles are ideal to answer these questions because these evolutionary transitions have occurred repeatedly in this group, leading to varied sex-determining mechanisms. Namely, reptilian sex determination encompasses TSD in crocodilians and tuatara, genotypic sex determination (GSD) in snakes, and either TSD or GSD in turtles and lizards (some lizards even combine GSD and TSD) [3]. Furthermore, TSD and GSD is not the same in all reptiles [3,7]. Rather, TSD reptiles respond to temperature in one of three patterns: (a) by producing males at colder and females at warmer conditions, (b) the opposite, or (c) by producing males at intermediate values and females above and below (reviewed in [7]). GSD reptiles also vary, as some possess female- and others male-heterogametic sex chromosomes, including homomorphic or heteromorphic ZZ/ZW, XX/XY, X_1_X_2_Y, Z_1_Z_2_W, and ZW_1_W_2_ systems [3,8]. Here, we concentrate on turtles, an emerging model group with growing genomic resources, and explore the karyotypic changes that accompanied the evolution of the genomic architecture of sexual development during transitions between TSD and GSD in this group. Hereafter, we will refer to XX/XY ZZ/ZW systems as XY and ZW, respectively.

## 2. Sex Chromosome Evolution

Theoretical models suggest that vertebrate sex chromosomes evolved from ESD or from polygenic sex determination [2] by co-opting an autosomal pair of chromosomes harboring a sex-determining locus. This initial step is expected to trigger a cascade of events that leads to the eventual degeneration of the heterogametic Y or W due to their unusual mode of transmission through a single sex [2]. The model proposes that first, recombination is reduced adaptively via chromosomal inversions or via selection on a modifier locus favoring recombination suppression [9], to preserve the linkage disequilibrium between the sex-determining locus and sexually antagonistic genes [10,11,12] (e.g., Y genes that favor males but are harmful if they were expressed in females [13]). Consequently, mildly deleterious mutations accumulate in the non-recombining region via Muller’s ratchet or genetic drift, causing Y or W genes to lose their function and ultimately disappear altogether [14]. Moreover, strong selection acting on the sex-determining region can induce background selection, genetic hitchhiking and selective sweeps that reduce genetic diversity in adjacent regions [2]. This degenerative process is prevented in the pseudo-autosomal regions of the sex chromosome where recombination remains intact [2]. Such extensive degeneration of the W and Y may constitute an evolutionary trap from which TSD evolution is difficult [15], because this transition requires traversing a valley in the fitness landscape (sensu [16]) where individuals are produced that carry suboptimal or lethal WW or YY genotypes. This problem may be averted when sex chromosomes are virtually homomorphic [2,17]. But do turtles follow this theoretical evolutionary trajectory? Valuable existing information sheds light on the evolutionary history of turtle sex chromosomes, despite how relatively little is known about their content compared to mammals or birds, partly because only a single GSD turtle genome assembly has been published (*Pelodiscus sinensis* [18]), and its sex chromosome scaffolds remain unmapped.

## 3. Sex Chromosomes were Gained and Lost Multiple Times in Turtles

**Figure 1:**
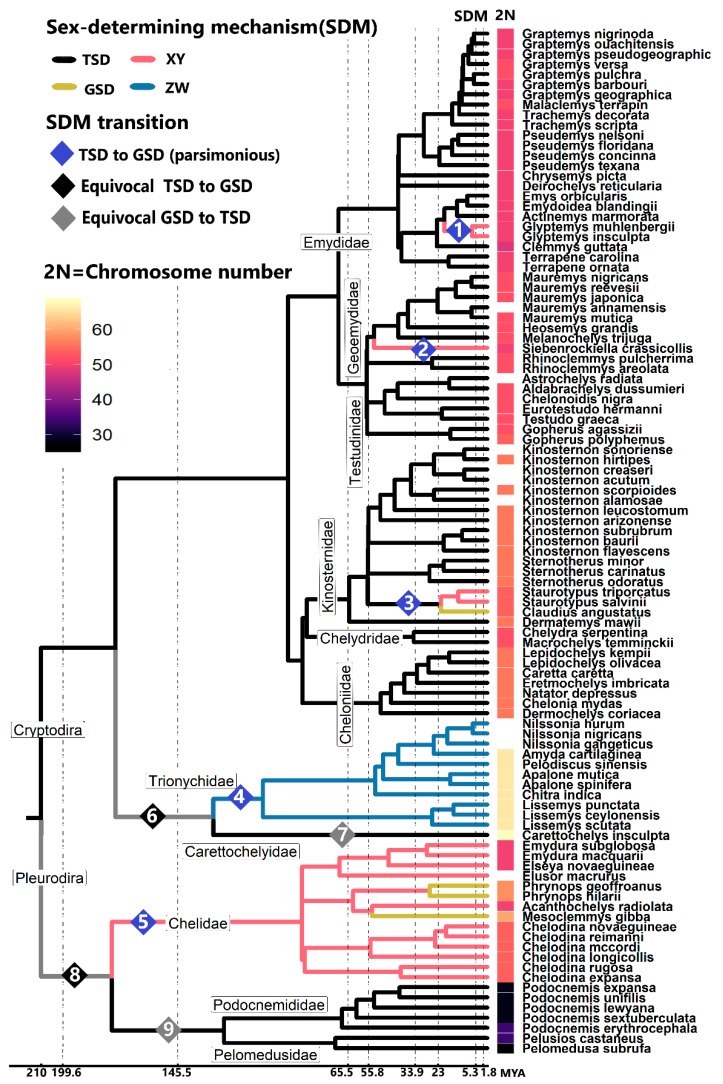
Phylogenetic relationships of turtles with a known sex-determining mechanism (SDM) and diploid number (2N). Timing of hypothesized gains and losses of sex chromosomes correspond to the timing of split of the colored branches. Diamonds indicate hypothesized transitions in sex determination. Transitions 1–5 are the most parsimonious. Transitions 6–7 and 8–9 represent alternative hypotheses to transitions 4 and 5, respectively, proposed based on ancestral reconstruction using maximum likelihood [19]. Data from [3,19,23,24,25,26].

Most turtles possess TSD, a system that appears ancestral to turtles, reptiles, and likely to all amniotes based on the most complete phylogenetic comparative analyses possible to date given the existing information [15,19,20,21]. These species-level phylogenetic analyses revealed that the history of turtle sex determination is marked by the retention of an ancestral TSD mechanism in most chelonian lineages, punctuated by few transitions to sex chromosomes (five so far identified), and two potential reversals from GSD back to TSD where sex chromosomes may have been lost [19,22] (Figure 1). However, these evolutionary transitions are not all created equal, and instead, they have followed unique trajectories accompanied by profound genomic modifications, as will be described.

**Figure 2:**
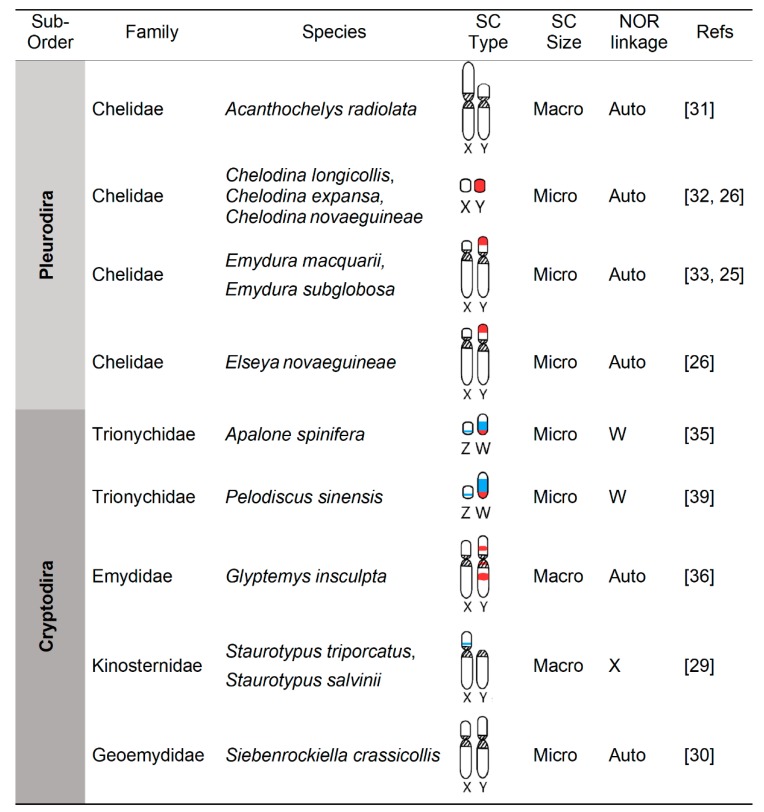
Karyotypic characteristics of turtle sex chromosomes (SC) from species studied cytogenetically. Macro = macro-chromosomes; Micro = micro-chromosomes; Streaked regions = centromeres; blue = nucleolar organizing region (NOR); red = male-specific regions in the Y or female-specific region in the W, detected via CGH; as described in the text and reviewed in [28].

Indeed, of the >355 recognized species of turtles to date [27], sex determination is known in only 101 of them. These include 31 GSD species, of which 27 have a characterized sex chromosome system that vary in age, heterogamety, homology, shape, and size (turtles possess macro and micro chromosomes [28] (Figure 1 and Figure 2). Four other GSD turtles were identified as such because they produce 1:1 sex ratios across incubation temperatures, thus ruling out TSD [4], but their heterogamety remains unknown. This gap exists because few studied turtles have large heteromorphic sex chromosomes easily visualized using classical cytogenetic techniques [29,30,31]. Thus, the detection of virtually homomorphic sex chromosomes in other turtles requires higher-resolution molecular cytogenetic approaches, such as comparative genome hybridization (CGH), which only became available recently [25,26,32,33,34,35,36]. Male or female heterogamety has been identified recently in some GSD turtles by PCR or qPCR amplification of molecular markers first developed in closely related taxa [23,24,37].

From the distribution of these sex chromosome systems in the turtle tree of life, male heterogamety was inferred to have evolved at least three times in the suborder Cryptodira (the turtles who hide their necks inside their shell) and at least once in the suborder Pleurodira (the turtles who bend their necks to the side outside their shell) (Figure 1). These events gave rise to the 16 turtle XY systems identified thus far: 11 in the pleurodirans within a single family Chelidae—*Acanthochelys radiolata, Elseya novaeguineae*, six in the genus *Chelodina,* and two in *Emydura*, [25,31,32,33], plus five from three cryptodiran families—*Siebenrockiella crassicollis*, two in *Glyptemys*, and two in *Staurotypus* [23,29,30,36,38].

Curiously, unlike the repeated independent evolution of XY in chelonians, a single origin of female heterogamety is known, in the cryptodiran family of softshell turtles (Tryonichidae). Indeed, ZW has been documented in 10 soft-shell turtles, two cases (*P. sinensis* and *Apalone spinifera*) by molecular cytogenetic data [35,39], and eight others by PCR/qPCR— three species in the genus *Lissemys*, three in *Nilssonia*, plus *Chitra indica* and *Amyda cartilaginea* [24]. The report of a non-softshell turtle (*Pangshura smithii*) having an independently evolved heteromorphic ZW was debunked recently, as it was due to a karyotyping error [40,41]. Therefore, it is unclear if *P. smithii* has GSD with homomorphic sex chromosomes or TSD. Similarly, the co-existence of sex chromosomes and TSD was empirically refuted in *P. sinensis* and *Chrysemys picta* [42,43], whereas thermal sex reversals occur in several lizards [44,45,46,47,48]. However, why XY systems are more likely to evolve in turtles than ZW systems is unclear, although this pattern matches theoretical models (reviewed in [2]). Some of these models predict that when sex chromosomes arise during the evolution of separate sexes in a population of hermaphrodites, selection favors first the spread of a recessive male-sterility mutation that produces females when homozygous, and then selection favors the spread of a dominant female sterility mutation in the hermaphrodites that produces males, leading to the formation of a XY system (reviewed in [2]). Alternatively, sexual selection, which is stronger in males in general, may favor the fixation of a major sex-determining factor beneficial to the heterogametic sex, thus favoring the evolution of an XY system (reviewed in [2]).

From a karyotypic perspective, the first difference that is noticeable between the 13 turtle sex chromosome systems for which cytogenetic data are available (Figure 2), is that five are micro-sex-chromosomes (μSC) (in the softshells *A. spinifera, P. sinensis* and in the Australian chelids of the genus *Chelodina*), whereas the other eight are macro-sex-chromosomes (MSC). The second notable karyotypic characteristic, is that four independent evolutionary events resulted in heterogametic sex chromosomes (Y or W) in turtles that are larger than the X or Z (in the genera *Emydura*, *Glyptemys*, *Siebenrockiella*, and in the family Trionychidae), whereas only two instances are known that resulted in a smaller Y than X (in the genera *Acanthochelys* and *Staurotypus*), and one case is known of the evolution of virtually homomorphic XY (in the genus *Chelodina*) (Figure 2). Cytogenetic data revealed that the larger size of the Y or W compared to the X or Z is due to the accumulation of repeat sequences, but the particular details vary among lineages. For instance, the repeats involved in this enlargement of the Y or Z encompass an expanded Z-linked NOR in trionychids [35], expanded heterochromatin (C- and G-bands) in the Y of *Glyptemys* [36] and *Siebenrockiella* [30], and accumulation of microsatellites in the Y of *Emydura* and *Elseya* [25,26,49]. Notably, the male-specific region of the *Emydura* Y likely evolved by an Y-autosome fusion that resulted in the translocation of an ancestral micro-Y chromosome present in *Chelodina* [25], which contributes to the larger Y in *Emydura*. This is the only case of Y-autosome fusion known in chelonians, whereas Y-autosome fusions are well documented in squamate reptiles and fish, and tend to be more common than X-autosome, Z-autosome, or W-autosome fusions [8]. Also remarkably, the larger size of *Staurotypus* X is not due to the degeneration of the Y but to the translocation of the NOR to the X, such that the Y in this turtle represents the ancestral condition and the X is the derived sex chromosome [50]. Taken together, the current data indicate that turtle sex chromosome differentiation does not always involve the shrinkage of the Y or W proposed in classical models of sex chromosome evolution, and add support to the alternative that sex chromosomes of varying degrees and type of differentiation are evolutionary stable states [1].

Despite the multiple gains of sex chromosomes identified in turtles, some evidence suggests that reversals back to TSD from GSD may have occurred also where sex chromosomes would have been lost, once in each chelonian suborder. In particular, two putative evolutionary reversals from GSD to TSD were identified by a maximum likelihood reconstruction of ancestral sex-determining mechanisms (SDMs): (1) one in the monotypic family Carettochelyidae (suborder Cryptodira) which split from the ZW softshell family Trionychidae in the Mesozoic ~148 Mya (http://www.timetree.org/), and (2) another in the superfamily Pelomedusoidea (suborder Pleurodira) which split from the XY family Chelidae at around a similar time ~144 Mya (http://www.timetree.org/) [19] (Figure 1). If true, this would mean that the TSD system in *Carettochelys insculpta* and Pelomedusoidea are secondary and independently derived traits, and might be expected to differ mechanistically from the ancestral TSD retained in most other turtles. However, these reversals are questionable given that their inference is not parsimonious [20], and that reanalysis of the same dataset by maximum likelihood reconstruction of ancestral SDMs using an updated software package did not recover the same result [21]. Yet, the significant intensification of the rate of molecular evolution of sexual development genes observed in both Carettochelyidae and Podocnemididae [22], above and beyond the already accelerated rate seen in their GSD sister lineages, supports the notion that GSD-to-TSD reversals might have taken place. Moreover, the branching of these lineages suggests that these reversals might have occurred soon after GSD evolved in the common ancestor of Carettochelyidae and Trionychidae, and in the common ancestor of Podocnemididae and Chelidae, likely before extensive differentiation of their sex chromosomes accrued that would have been harder to overcome [1,15]. Nonetheless, the support for these putative reversals remains relatively scant and stronger inferences require further research.

## 4. Independent and Convergent Evolution of Turtle Sex Chromosomes

The existing data indicate that not all turtle sex chromosomes are homologous to each other [28]. Indeed, partial data on the gene content of the sex chromosomes of some cryptodiran turtles for which information is available (*Staurotypus triporcatus*, *Glyptemys insculpta*, *Siebenrockiella crassicollis,* and softshells such as *A. spinifera* and *P. sinensis*) revealed that the evolution of their XY and ZW chromosomes occurred by the co-option of different ancestral reptilian autosomes [28]. Yet, these turtle sex chromosomes may share a deeper homology with blocks of a more ancient proto sex chromosome [28]. In particular, gene content indicates that *S. triporcatus* XY share homology with chicken’s Z (GGA-Z) and the ZW of *Gekko hokouensis* lizards, and with a block of the frog *Xenopus tropicalis* chromosome 1 (XTR1) [28,34,51]. This XTR1 block contains *Dmrt1*, chicken’s sex-determining gene, whose action is dosage-dependent [28,51]. On the other hand, the ZW of softshells (e.g., *A. spinifera* and *P. sinensis*), which derive from a single gain in the common ancestor of the softshell family *Trionychidae* [24,35], are homologous to each other, to chicken GGA15, and to the X of *Anolis carolinensis* lizards [28,38], and share partial homology with a second block of XTR1 [28].

In yet another evolutionary twist, the only two turtle lineages known to have recruited the same pair of ancestral autosomes, *G. insculpta* and *S. crassicollis*, did so independently [28]. Indeed, the XY in these two species are homologous to each other, to GGA5, and to XTR5, which contains the male development gene *Wt1* [28,36]. The notion that these two XY systems represent convergent evolution and followed independent trajectories is also supported by a secondary homology shared between the short arm of *G. insculpta* (but not *S. crassicollis*’) XY and GGA26, which surprisingly, is homologous to a third block of XTR1 [28,36]. To make matters even more intriguing, another (fourth) block of XTR1 shares homology with the sex chromosomes of other vertebrates, namely, the X of therian mammals including humans, and the Z of the lizard *Takydromus sexlineatus* [28,52]. Moreover, XTR1 is also homologous to the sex chromosomes of three other anuran amphibians [53].

Thus, the origin of these independently derived sex chromosomes appears to be non-random. Instead, all the data combined suggest that certain ancestral chromosomes constitute proto-sex chromosomes that are more likely to take on a role as sex chromosomes in various derived taxa (e.g., XTR1 or a putative ancestral XTR1 + XTR5 from which many turtle chromosomes derive) [28]. It is noticeable that a common ancestral chromosome can follow evolutionary trajectories leading to both male or female heterogamety (e.g., softshell turtles ZW versus *Anolis* lizard XY; *Staurotypus* turtles XY versus chicken’s and *Gekko* lizards’ ZW, etc.). Perhaps chromosomes which are rich in genes involved in sexual development are better suited to take on a role as sex chromosomes, and perhaps both the evolution of these sex chromosomes and their fixation in populations may be facilitated by genomic rearrangements [12]. Additionally, chromosomes with genes resilient to changes in dosage may also be favored for co-option as sex chromosomes as they may be buffered naturally from the degeneration and gene content depletion that may be inevitable in the evolving Y or W [28,54,55].

Taken together, all evidence permits drawing parallels and contrasts between the sex chromosomes of chelonians and other reptilian lineages. Namely, like other reptiles, turtle sex chromosomes vary in the degree of heteromorphism (Figure 2), and some of them carry the genes of the nucleolar organizing region (NOR) [28]. Just like turtles, both lizards and snakes co-opted various ancestral autosomes as sex chromosomes independently [56,57,58]. Unlike turtles, however, the repeated evolution of sex chromosomes in squamates (lizards and snakes) occurred not only as transitions from TSD but also as transitions between male and female heterogamety, a process that has not been detected in turtles. For instance, the recent discovery of convergent XY sex chromosomes in pythons and boas refuted the long-held notion that they share the ZW system previously thought to be ubiquitous and homologous across snakes [57]. Also, no case of multiple sex chromosomes is known in turtles, whereas examples exist in squamates [3,58].

## 5. Ecological and Karyotypic Correlates of the Birth and Death of Turtle Sex Chromosomes

What are the causes and consequences of the gain or loss of turtle sex chromosomes? The answer to this question is not fully resolved, yet significant strides have been made towards solving this mystery. Some ecological and karyotypic correlates of sex chromosome evolution exist in turtles, including diploid number, climate change, population sex ratios, and longevity. In particular, turtles exhibit high variance in the diploid number of chromosomes ranging from 2N = 28 to 68 (Figure 1). The genomic rearrangements responsible for generating this diversity occurred at a rate 20× higher in turtle lineages that underwent an evolutionary transition in their sex-determining mechanism than in lineages whose sex determination remained static [19]. However, which is the cause and which the consequence remains unclear. For instance, perhaps the chromosomal rearrangements responsible for changes in chromosome number are fertile grounds for changes in sex determination, as they could affect gene expression through direct disruption of genes, changes in regulatory elements or altered positional effects [59]. Further, while chromosomal fusion or fission are large-scale changes that alter chromosome number, smaller-scale rearrangements can also have significant ramifications. For example, inversions induce the repression of recombination between homologs of a chromosomal pair, which is a hallmark of sex chromosome evolution [60]. Such inversions are known in turtles, and include multiple parts of the male-specific region of Y chromosome in *G. insculpta* that contains the male development gene *Wt1*, which might have facilitated the transition from ESD to GSD in this lineage [36]. But some other identified inversions and translocations involving sexual development genes (*Dax1*, *Fhl2*, *Fgf9*, *Sf1* and *Rspo1*) across TSD and GSD turtles do not correlate with transitions in sex determination [61]. Alternatively, perhaps transitions in sex determination trigger molecular changes that render the genome unstable and more susceptible to chromosomal fusion or fission which, in turn, will alter the chromosome number [19]. These pending questions remain the foci of active research.

Climate change is an ecological factor that may trigger such transitions in sex determination in turtles, particularly, the evolution of sex chromosomes from TSD, as an adaptive response to alleviate extreme biased sex ratios caused by steady warming or cooling events [19,62]. Specifically, a theoretical model proposes that ESD could be adaptive in species inhabiting patchy or heterogenous environments where some environments increase the fitness of one sex and other environments increase the fitness of the other sex (provided that offspring cannot predict the environment they enter – nor can their parents, and that individuals from all environments mate at random) [63]. Under these circumstances, the plasticity provided by ESD permits individuals to develop into the sex that is better suited in each environment. However, if environments become homogenous, or in the case of TSD, if climate change becomes directionally warmer or cooler over time, population sex ratios may be skewed. Then, natural selection could favor the evolution of sex chromosomes via the evolution of a masculinizing or a feminizing locus, resulting in a XY or ZW system, respectively [2], which will restore sex ratios closer to parity. (This model assumes that 1:1 sex ratio is optimal, which is not always the case [62,64,65]. This scenario is supported in turtles by the observation that some lineages known to have experienced a transition in sex determination split from their sister lineages at geological times when global temperatures were near a peak [19]. However, relatively few transitions in sex determination are documented in turtles across repeated bouts of past climate change compared to squamates, the other reptilian clade with labile sex determination. This difference may be attributed to their contrasting life history, particularly their differences in lifespan [21]. Indeed, the greater longevity of turtles would enable them to withstand longer stretches of biased sex ratios caused by directional climate change compared to lizards and snakes, thus explaining the frequent retention of TSD in turtles and the evolution of GSD more readily in squamates [21]. However, the speed of contemporary climate change and the predicted warmer average temperatures that fluctuate more widely pose a challenge to extant TSD turtles in unprecedented ways [66,67].

## 6. The Architecture of Sex Determination with and without Sex Chromosomes

The regulatory gene network that underpins the development of males and females is composed of many common elements across vertebrates, but TSD and GSD lineages differ in the level of plasticity of this regulation to environmental inputs [68,69]. Indeed, the signal which initiates the cascade of events leading to sex differentiation is hard coded in the genome within the sex chromosomes in the form of a master sex-determining gene(s), whereas in TSD this developmental decision is triggered by an environmental factor (i.e., temperature) [4]. Thus, because sex chromosomes contain the only consistent genomic differences between the sexes, and reptilian sex chromosomes are diverse, their study illuminates the evolution of master sex-determining genes, and more generally, our understanding of the genetic architecture underlying sexual development in vertebrates. For instance, in therian mammals, the Y-linked *Sry* initiates male differentiation [70], whereas sex determination in birds relies on the dosage of the Z-linked gene *Dmrt1* in ZZ-males versus ZW-females [71]. However, no master sex-determining gene(s) has been discovered in reptiles. Some candidates exist in reptiles, including in chelonians [72,73,74,75,76,77], and the list is growing thanks to the advent of third generation sequencing and advanced molecular cytogenetics (e.g.,) [28,61,74,75,76,77,78]. Namely, the XY of *S. triporcatus* turtles contains *Dmrt1* [28,78], a gene whose molecular evolution is linked to transitions in sex determination in reptiles [79] and which displays sexually dimorphic expression in TSD turtles ([80] and references therein). Notably, while *Sry* is exclusive to therian mammals, *Dmrt1* or its orthologs or paralogs are master triggers of sex determination in various vertebrates (reviewed in [80]). However, whether *Dmrt1* is the master sex-determining gene in *Staurotypus* is untested. Nonetheless, *Dmrt1* retention in both the X and Y in *S. triporcatus* and in the Z and W of *G. hokouensis* lizards [28] indicates that *Dmrt1′*s mode of action likely differs between birds and these reptiles. *Dmrt1* was demonstrated to be important in testicular differentiation of the GSD softshell *P. sinensis* [81], but its autosomal nature [61] invalidates its potential role as a sex-linked master sex-determining gene. Another example of a putative master sex-determining gene in GSD turtles is *Sf1*, a male-development gene that translocated to the ZW of *Apalone* softshells lineage from its ancestral autosomal location [61]. *Sf1* was proposed as a potential candidate for this role based on its monomorphic expression by temperature in *Apalone* softshell turtles, in contrast to its differential expression at male- versus female-producing temperature in the TSD turtle *C. picta* [72,73,76]. A third candidate comes from the XY of *G. insculpta* and *S. crassicollis* turtles, which contain the male development gene *Wt1* [28,36], a transcription factor whose relic thermosensitive expression in early embryos of *Apalone* is countered by the immediate downstream action of *Sf1* [82,83]. Noticeably, the relic thermosensitive expression in *Apalone* of another important sexual development gene, *Dax1*, is also countered by the immediate downstream action of *Sf1* [83].

The identification of top regulators of sexual development remains even more elusive in TSD turtles, because no genomic differences exist between the sexes, and finding candidates requires the analysis of the response of gene regulatory pathways and networks to incubation temperature [82]. Candidate gene approaches, along with transcriptome and methylome analyses, plus initial functional assays have provided a number of candidates in TSD turtles for an upstream function in sexual development (e.g., [74,75,76,77], ruling out elements whose sexually dimorphic expression occurs too late in development to act as top master regulators (e.g., [73,76,82,83,84,85,86,87,88,89]). To complicate matters further, this body of evidence has uncovered significant divergence in the transcriptional patterns of genes in this regulatory network [73,80]. As this growing body of work cannot be summarized properly in the limited space available here, we direct the reader to some excellent reviews on this area (e.g., [68,90,91]), and highlight here only some salient results. Comparative data from *C. picta* (TSD) and *Apalone mutica* or *A. spinifera* (GSD) have provided important insights. In particular, early dimorphic transcription in *C. picta* of genes responsible for the formation of the bipotential gonad and later testicular development in vertebrates (*Sf1* and *Wt1*), rendered these elements as potential activators of *C. picta*’s thermosensitive period (when temperature exerts its strongest effect on population sex ratios) [72,73,82]. Their early expression in *C. picta* coincides with the time when the expression of genes involved in chromatin organization and chromatin modification is enriched [92,93] but also with the dimorphic expression of female-development regulators, such as *Ctnnb1* (*β-catenin*) and its downstream target *Fst* [93]. On the contrary, the later differential expression of *Dax1* [83], *Dmrt1* [80], *Sox9* and *Aromatase* [73] ruled them out for a role as upstream TSD thermal sensors/activators in *C. picta*. An interesting candidate TSD gene is *CIRBP*, which exhibits allelic-specific expression at male- and female-producing temperature in *Chelydra serpentina* turtles (TSD) [75], but which shows thermo-insensitive expression in *C. picta* and *A. spinifera* [93]. Epigenetic regulation of TSD also appears important. For instance, DNA methylation regulates *Aromatase* in *Trachemys scripta* turtles, a key ovarian development gene that is also affected by histone modifications [94,95]. Methylome analysis indicated that some of the thermosensitive responses of the regulatory network of sexual development in TSD turtles is mediated by DNA methylation of additional components other than *Aromatase* [92,96]. And other epigenetic modifications also influence sexual development. For instance, the histone demethylase KDM6B induces the transcription of *Dmrt1* in *T. scripta* [89], a gene important in testicular formation during the thermosensitive period in this turtle [87]. Furthermore, transcriptomic analysis of epigenetic machinery genes suggests that TSD, at least in *C. picta*, is potentially mediated by hormonally controlled epigenetic processes, or by epi-genetically controlled hormonal pathways (via acetylation, methylation, and ncRNAs) [77]. Importantly, the response of the epigenetic machinery genes to temperature indicate that differences in key epigenetic events before the onset of the thermosensitive period may define the divide between TSD and GSD, as represented by *C. picta* and *A. spinifera* [77]. It should be noted that while these growing efforts are helping to resolve the position of these factors in the sexual development cascade, further research is still needed.

## 7. Consequences of Sex Chromosome Evolution—Dosage Compensation and Faster Molecular Evolution

The degeneration of the heterogametic sex chromosomes Y and W over evolutionary time [97,98] described earlier can have ill fitness consequences unless these effects are balanced by the evolution of counter mechanisms. For instance, in human, chicken, and multiple animal species with sex chromosomes, only a small fraction of genes remain active in Y or W compared to X or Z [99,100]. This loss of function or physical loss of genes in the Y or W generates a gene dosage imbalance between autosomes and sex chromosomes, and between males and females (i.e., the homogametic sex will have twice as much dosage of X- or Z-genes compared to the heterogametic sex) [2]. However, the balance of dosage is very important for genes to maintain their proper function as they are part of genetic networks. An adaptive solution to this problem evolved in the form of dosage compensation mechanisms that modify the transcription of genes with differential dosage so that their expression is balanced fully or partially, either along the entire sex chromosome (global dosage compensation) or only for some important genes (local dosage compensation) [2,14,101,102].

Earlier animal data suggested that global and complete dosage compensation is more predominant in XY taxa (such as therian mammals), while local and partial dosage compensation is more common in ZW taxa (such as birds), but numerous exceptions discovered over time called this conclusion into question [101,102,103]. Very little is known about dosage compensation in reptiles, with only three studies published on squamates and none on turtles thus far, despite the fact that the diversity of independently evolved sex chromosomes in closely related reptiles renders them an ideal group to address this issue. Existing reptilian data indicate that *A. carolinensis* lizards utilize a mixed pattern of dosage compensation [104]. Namely, some regions of the X chromosome in *A. carolinensis* show complete dosage compensation and some regions lack complete compensation, indicating that the evolution of complete dosage compensation might be an ongoing process [104]. On the other hand, incomplete dosage compensation is seen in two ZW squamates, i.e., snakes and the Komodo dragon [56,105].

Sex chromosomes and autosomes also differ in ways that influence the rate of divergence of sex-linked genes compared to their autosomal counterparts. Specifically, the Y or W are hemizygous, experience reduced recombination and are inherited through one sex only, whereas the X or Z are inherited through both sexes but spend twice as much time in one sex [1,2]. These differences lead to striking differences in their molecular evolution. Indeed, faster-Z and faster-X divergence is expected because reduced recombination of sex chromosomes facilitates the accumulation of beneficial mutations and the removal of deleterious mutations at higher rates than in autosomes, and because their smaller population size renders them more susceptible to genetic drift [106,107,108]. While faster rates of divergence of coding sequences are documented in X and Z of many species, including birds (e.g., *Gallus gallus*, *Taeniopygia guttata*), mammals (*Mus castaneus* and *Homo sapiens*) and fruit flies (*Drosophila melanogaster*) [107,108,109,110,111,112], research in reptiles is in its infancy. Namely, initial studies in turtles examining a very small set of sex-linked genes detected faster-Z in *P. sinensis* but not in *A. spinifera* (both softshell turtles), and slower-X in *S. triporcatus* turtles compared to orthologs in sister taxa where these genes are autosomal [76]. Additionally, a comparison of turtles, crocodilians, squamates, birds and mammals, detected three genes (*Dmrt1*, *Ctnnb1, Ar*) with faster amino acid substitution rates when they are Z-linked (*Dmrt1* and *Ctnnb1*, Z-linked in birds and snakes) but not when they are X-linked (*Ar*, X-linked in mammals), compared to when they are autosomal [22]. Similar to turtles, faster-Z is observed in chicken and other birds [107] whose sex chromosomes are homologous to *Staurotypus* X, and faster-X is supported in *A. carolinensis* [104] whose sex chromosomes are homologous to *Apalone*’s Z. On the contrary, the report of faster-Z in snakes [56] needs revisiting, since it is based on data from *Boa* whose purported ZW chromosomes are now known to be autosomes [57]. The major roadblock to study molecular evolution of sex chromosomes in turtles and other reptiles should be alleviated with the publication of additional chelonian genomes with mapped sex chromosomes.

## 8. Conclusions

Reptiles have diverse systems of sex determination as well as sex chromosomes which are unmatched in mammals and birds. Although sex chromosomes and sex determination in many species of reptiles have been studied, the genomic basis of sexual development has yet to be fully characterized in reptiles. Turtles represent a clade where multiple sex chromosomes evolved independently (XY and ZW) from ancestral TSD systems (or were perhaps lost during GSD to TSD reversals), giving rise to sex chromosomes of with varying size, age and homology. Unlike lizards, there is no evidence reported for the influence of environmental factors overriding sex chromosomes in GSD turtles. We argue that the characterization of sex chromosomes and sexual development in GSD turtles might be the key to identifying major players of sexual development including the master sex determining gene(s) in reptiles. Convergent evolution of sex chromosome in *G. insculpta* and *S. crassicollis* where XY systems evolved independently from the same ancestral autosome would offer a great opportunity to identify genes that are co-opted as sex determining genes. Taken together, existing data indicate that turtles support some tenets of classic theoretical models of sex chromosome evolution, while other tenets are countered. For instance, the evolution of some turtle sex chromosomes involves chromosomal rearrangements such as the translocation of sexual development genes, or inversions that may contribute to their divergence, yet that divergence does not always result in a morphologically degenerate Y or W. Finally, advanced technologies like whole genome sequencing, transcriptomics, methylomics, and other epigenetic approaches should improve our understanding of chromosomal structure and content, global and local gene expression and epigenetic signatures, all of which will be vital to decipher the enigmatic evolutionary trajectories of sex chromosomes and to develop a model of sexual development in turtles, reptiles, and vertebrates.
